# Rapid Detection of Soil Available Phosphorus using Capacitively Coupled Contactless Conductivity Detection

**DOI:** 10.2174/0115701794295930240902050855

**Published:** 2024-09-23

**Authors:** Jun Gao, Wei Li, Jiaoe Li, Rujing Wang

**Affiliations:** 1 University of Science and Technology of China, Science Island Branch, Graduate School of USTC, Hefei, 230026 ,P.R. China;; 2 Intelligent Agriculture Engineering Laboratory of Anhui Province, Institute of Intelligent Machine, Hefei Institutes of Physical Science, Chinese Academy of Sciences, Hefei, 230031, P.R. China

**Keywords:** Soil available phosphorus, on-site rapid pretreatment, detection, capacitively coupled contactless conductivity detection, capillary electrophoresis, back propagation neural network model

## Abstract

**Background:**

In China, the traditional method for analyzing soil available phosphorus is inadequate for large-scale soil assessment and nationwide soil formulation demands. To address this, we propose a rapid and reliable method for soil-available phosphorus detection. The setup includes an on-site rapid pre-treatment device, a non-contact conductivity detection device, and a capillary electrophoresis buffer solution system composed of glacial acetic acid and hydroxypropyl-β-cyclodextrin.

**Methods:**

The on-site rapid pre-treatment process includes fresh soil moisture content detection (moisture rapid detector), weighing (handheld weighing meter), stirring (handheld rapid stirrer), and filtration (soil rapid filter) to obtain the liquid sample, and direct injection (capillary electrophoresis detector). The phosphate ion detection parameters include capillary size, separation voltage, injection parameters, and electric injection. We used Liaoning brown soil, Henan yellow tidal soil, Heilongjiang black soil, and Anhui tidal soil as standard samples. Additionally, we used mathematical modeling methods and machine learning algorithms to analyze and process research data.

**Results and Conclusion:**

Following calibration with standard samples, the experimental blind test samples demonstrated conformity with the national standard method, exhibiting a relative standard deviation of less than 3%. The proposed pre-treatment device and non-contact conductivity detector are powered by lithium-ion batteries, rendering them ideal for extended field operations. The non-contact conductivity detector obviates the need for direct contact with test samples, mitigating environmental pollution. Furthermore, the neural network model exhibited the highest level of goodness of fit in chemical data analysis.

## 1 INTRODUCTION

The conventional approach to analyze soil available phosphorus typically demands significant manual effort, material resources, and time, rendering it unsuitable for nationwide large-scale soil formulation and comprehensive soil assessment [1-4]. Existing methods are typically designed for laboratory conditions. The pre-treatment methods, such as the Olsen method [5], the Bray-1 method [6] and the Mehlich-3 method [7], rapid ASI combined leaching method [8], molybdenum and antimony colorimetric method [9], often requiring long detection cycles and complex operations, making them unsuitable for rapid, low-cost farmland detection [10]. Moreover, commonly used spectral detection methods, such as visible/near-infrared, laser-induced breakdown spectroscopy [11], the use of calibration techniques to reflectance spectra for analysis [12], modeling, and fitting with the true value to predict soil nutrient data [13], are susceptible to soil texture, moisture, color, surface roughness, *etc*. [14]. Ion adsorption and ion-selective electrode methods [15] commonly use ion exchange resins to capture and adsorb nutrient ions from the soil, collect them, and then employ ion-selective electrodes to directly detect the leachate. However, these methods are susceptible to interference from the complex environmental conditions of the soil system [16]. When electrodes are easily poisoned, their performance degrades, leading to reduced accuracy [17]. Microfluidics offers advantages such as high sensitivity, rapid real-time analysis [18], reduced sample and reagent consumption, portability, and potential for integration and automation in the soil testing for molecular imaging probes [19]. However, it also faces challenges including complexity in design and operation, the need for extensive calibration and validation, fragility, high initial setup costs, and scalability issues for large-scale testing. Additionally, portable detection systems have garnered significant attention, especially in agricultural and food safety contexts [20]. A portable system using on-site soil pH and potassium detection, utilizing 3D printed sensors [21] and a PSoC4 microcontroller [22], eliminates the need for lab equipment and pre-treatment. Paper-based lab-on-a-chip (pLOC) devices enable rapid contaminant detection *via* various fabrication methods and detection techniques. Integration of ICT tools enhances real-time monitoring and traceability in food supply chains. Portable pLOC devices provide rapid on-site contaminant detection and can integrate with smartphones [23].

Therefore, developing a pretreatment and detection method with minimal pretreatment time, rapid detection speed, and low operational costs is an imminent challenge in achieving swift on-site soil pretreatment and detection. To tackle this challenge, we propose an approach for rapid, reliable soil available phosphorus detection. Soil undergoes pretreatment *via* immersion in ultrapure water [24], and the resultant soil detection solution is directly transferred into portable pretreatment equipment, and then fed into capillary electrophoresis detection equipment. This streamlined process significantly simplifies automated soil nutrient analysis. The non-contact conductivity detection electrode forms a coupling capacitance between the insulating layer and the solution in the pipe [25]. The solution between the two electrodes constitutes an equivalent resistance. By applying a certain frequency of AC signal at the excitation electrode, the detection electrode captures a current signal reflecting the solution's conductivity. In the capillary electrophoresis non-contact conductivity detection device, the electrode does not directly contact the solution, thereby preventing issues such as polarization and corrosion [26]. The simple structure makes this electrode well-suited for soil nutrient analysis processes [27]. The capillary electrophoresis non-contact conductivity detection device tackles issues such as electrode polarization and corrosion under its non-contact design with the solution. With its simple structure, easy maintenance, and robustness, this electrode is well-suited for large-scale, on-site rapid detection of available soil phosphorus [28].

In the later section of the article, focused on chemical data modeling and analysis, our objective is to streamline the experimental process through mathematical modeling [29]. Typically, determining soil available phosphorus content requires numerous repetitions to ascertain crucial parameters, resulting in a cumbersome experimental procedure [30]. Furthermore, since Ct values often depend on the results of multiple trials, the conventional experimental approach not only consumes time and effort but also carries the risk of human error, thereby compromising both efficiency and accuracy. To mitigate these challenges, we introduced mathematical modeling methods [31] and machine learning techniques [32] to enhance the efficiency and accuracy of the soil available phosphorus determination. By establishing mathematical relationships among V1, S1, and Ct, we can better grasp intricate dynamics among these parameters and achieve precise predictions of *C_t_*. Concurrently, machine learning algorithms bolster the modeling and prediction capabilities of complex soil systems by assimilating vast amounts of experimental data, providing a novel avenue for swift and accurate *C_t_* determinations.

## 2 MATERIALS AND METHODS

### 2.1 Basics of Ion Electrophoresis Separation in Capillary

Under the action of an electric field, ions move in a capillary tube and are subjected to the combined action of the electric field force *F_E_* and the solvent resistance *F_f_*. After a period of time, they reach a constant speed, and enter a steady state. According to the law of electricity, the electric field force is the product of the ion charge *q* and the electric field strength E: *F_E_ = qE*. According to fluid mechanics, the flow resistance of spherical ions *F_f_* is proportional to their effective radius r, speed of movement *v*, and the viscosity of the solvent η. The specific details are provided in Equation (1) [33].


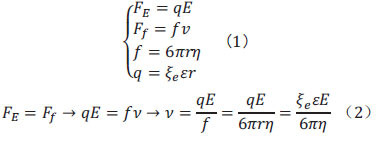


When the electric field force and friction force are in balance, *v* can be derived from Eq. (2).

Description: *F_E_*: friction; *F_f_* : solvent resistance; *E* : electric field strength; *q* : ion charge; *f* : coefficient of friction; *v* : migration speed of lysogenic particles in electric field; *ξ_e_* : charged ion zeta potential; *ε* : solution dielectric constant; *η* : medium viscosity; *r* : effective radius of ion.

The migration speed *v* of charged particles in the electric field is related to the following parameters: *E* (electric field strength); *η* (medium viscosity); *ε* (solution dielectric constant); *ξ_e_* (charged ion zeta potential); *r* (effective radius of ion): In the separation buffer solution of soil available phosphorus, the size and shape of the phosphate ion, the effective charge number, quality, and other parameters are different from the nature of the constituent materials of the buffer system, which constitute the basis for separating the phosphate ion in capillary electrophoresis.

### 2.2 Separation Efficiency

The separation efficiency in capillary electrophoresis is quantified by the number of theoretical plates (N), a concept derived from chromatographic theory and expressed by the Giddings equation as 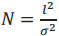 (*l*: migration distance, *σ*^2^: variance of the concentration distribution in the zone) [34]. When two components must be completely separated (resolution Rs=1) in Eq. (3),


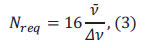


where *Δν* is the difference in migration speed of two components, 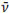 is the average migration speed. The capability of an electrophoresis system to resolve components with similar mobilities is its resolution [35]. This degree of separation can be expressed using dimensionless numbers for components i and j, according to the Giddings equation, as the resolution *Rs* for components i and j by Eq. (4).


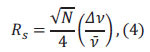


where 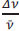 is the relative speed difference between two adjacent components (*i*, *j*).

In an ideal scenario, where the electroosmotic flow (EOF) is not considered, and 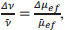 the resolution can be expressed as Eq. (5).


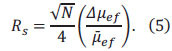


However, in practical situations with EOF present, the apparent mobility 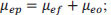 Theoretical plates, 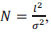 and N can be deduced from Eq. (6).


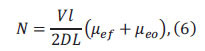


*Rs* can be derived from Eq. (7).


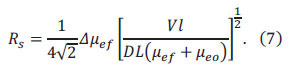


where several factors influence separation efficiency, V (applied voltage), with higher electric fields yields greater separation efficiency, l/L (effective column length ratio), with larger ratios enhancing resolution, *Δ*μef** (electrophoresis effective mobility difference), where solutes with smaller diffusion coefficients exhibit higher separation efficiency, *μ_eo_* (EOF mobility), with higher EOF speeds reducing the solute residence time of solutes in the capillary and increasing separation efficiency.

For the detection of inorganic ions, like soil available phosphorus and other nutrients, their relatively large molar conductivity requires a low molar conductivity background electrolyte for buffer design [36]. For instance, the common MES/His buffer uses the ampholyte histidine; for high ionic strength without substantially increasing current, benefiting conductivity detection. Buffer design should balance high separation efficiency and low Joule heat. The glacial acetic acid/hydroxypropyl-β-cyclodextrin buffer ensures phosphate ion resolution and mitigates Joule heat. A 54 cm total capillary length with a 40 cm effective column length further guarantees phosphate ion resolution. We screened the optimal voltage within the negative high voltage range of 10 kV to 14 kV, finding 14 kV to provide the best phosphate ion resolution for the buffer system.

### 2.3 Basics of Capacitively Coupled Contactless Conductivity Detector Detection

The capacitively coupled contactless conductivity detector consists of an AC excitation voltage source, detection pool, and signal processing circuit. The excitation electrode emits a coupled excitation signal to the solution through the polyimide outer wall of the quartz glass tube, received by the detection electrode. The sample is judged by identifying signal differences from the sample background buffer. Under AC voltage, the detection cell is equivalent to a series resistor (solution resistance in capillary) and electrode capacitance (coupling electrode and capillary wall), in parallel with leakage capacitance (air coupling capacitance of detection electrode). Various detection parameters control electrode capacitance and leakage capacitance, achieving micro-molar level detection limits. The AC voltage frequency also greatly influences the response signal in C4D. The best response frequency relates to the detection cell (electrode length, spacing), buffer solution conductivity, and electronic circuit use [37]. The structure of the detection cell is closely related to the length and spacing of the detection electrode. The detection electrode directly affects the amplitude of the detection signal and the volume of the detection cell. At the same time, the detection sensitivity and separation efficiency are also related to the effective electrode length and spacing. In general, long electrodes achieve high sensitivity at low frequencies, while short electrodes provide better separation efficiency at high frequencies [38]. We used existing standard fused silica capillaries with 360 µm O.D. and 50 µm I.D. as the electrophoresis channel. The design of the detection cell, the length of the excitation electrode, and the receiving electrodes are 10 mm ring cylinders, the electrode spacing is 2 mm, the shielding electrode is 0.5 mm, and the tight joint is filled with conductive silver glue. The scheme of using a Colpitts oscillator circuit with a high-frequency transformer generates a sine wave signal with a maximum amplitude of 160 V and a maximum frequency of 200 kHz. Through repeated debugging and optimization of the designed excitation circuit, the 80 V/136 kHz sine wave is the best excitation signal for soil-available phosphorus [39].

## 3 TEST AND ANALYSIS

### 3.1 On-site Rapid Pretreatment Device and Process

As shown in Fig. (**1**) the soil on-site rapid pretreatment device consists of the following parts: (1) 3D printed soil moisture fast detector (independent design and processing); (2) 3D printed soil solution quick filter (independent design and processing); (3) Handheld detector; (4) Soil weighed meter; (5) Soil quick mixer. The above equipment is powered by a lithium battery and is suitable for manual pretreatment of the on-site soil.

Referring to the national soil standard pretreatment process, the specific on-site rapid pretreatment process is shown in Fig. (**2**)

Soil available phosphorus content *P*(*mg/kg*) is denoted by Eq. (8).


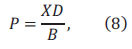


Where X(*mg/L*) is the detection value of the electrophoresis instrument; D(*mL*) is the sum of the solution volumes of A(*mL*) and C(*mL*), where A is the volumes of fresh soil moisture content, and C is the volumes of ultrapure water; *B*(g) is the fresh soil quality.

### 3.2 Buffer Solution System

For buffer solution preparation, 0.27 g of hydroxypropyl-β-cyclodextrin (HP-β-CD) was added to a 100 mL volumetric flask containing ultrapure water (18.2 MΩ•cm resistivity) and fully dissolved. Then, 150 μL of glacial acetic acid was added, and the solution was diluted to a final volume of 100 mL.

Standard phosphate ion configuration, potassium dihydrogen phosphate reagent (Aladdin Bio-Chem Technology Co., LTD), ultrapure water dilution, and plastic bottles were used for storage.

Standard soil samples, including Liaoning brown soil, Henan yellow tide soil, Heilongjiang black soil, and Anhui tide soil, were obtained from the Institute of Geophysical and Geochemical Exploration, Chinese Academy of Geological Sciences, which is a soil standard material accreditation agency in China.

### 3.3 Testing Parameters and Process

Capillary specifications are 45cm×50μm i.d., Leff =40 cm. Phosphate ion detection parameters included separation voltage, negative high pressure, -14 kV, injection parameter, -12 kV; electric sample injection, and injection time, 10 s with soil extractant; ultrapure water (18.2MΩ•cm resistivity).

Soil to water extraction ratio was 1:10 (1 g soil sample was extracted with 10 mL ultrapure water). The stirring time was 15 minutes. The filter parameters included a 0.45 μm pore diameter, and the filtration was conducted using a filter cake and syringe pressure filtration method. The sample detection process is shown in Fig. (**3**).

Among them, the detection cycle of a sample is as follows: the first injection is the unknown sample to be tested, obtaining the peak and peak area; the second time, a fixed concentration and volume of a known phosphate solution is added to the injection pool of the first sample to be tested, obtaining the second peak and peak area. Through two different peak and peak areas simultaneously, the phosphate concentration value of the unknown sample is converted to calculate the soil available phosphorus value [40].

The test sample solution concentration *C_t_* (*mg/L*) is related to the following parameters, corresponding to the electrophoresis peak area *S_1_*, after adding the standard solution, the target ion concentration in the test bottle was 
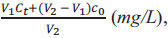
 corresponding to electrophoretic peak area *S_2_*. *Ct* can be derived from Eq. (9)


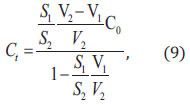


where *V_1_* (*mL*) is the solution volume before adding standard; *V_2_* (*mL*) is the solution volume after adding standard; *S_1_* is the electrophoresis peak area before adding standard; *S_2_* is the electrophoresis peak area after adding standard; *C_0_* (*mg/L*) is the initial concentration of the standard solution.

Then the ionic content of the soil sample is converted to *C* (*mg/kg*) by Eq. (10).


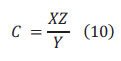


where *X* (*mg/L*) is the ionic content of the test soil sample; *Y*(*g*) is the soil quality leached by weighing the soil sample; *Z* (*mL*) stands for the volume of ultrapure water.

### 3.4 Soil Available Phosphorus Detection Analyzer

**Fig. (4):**
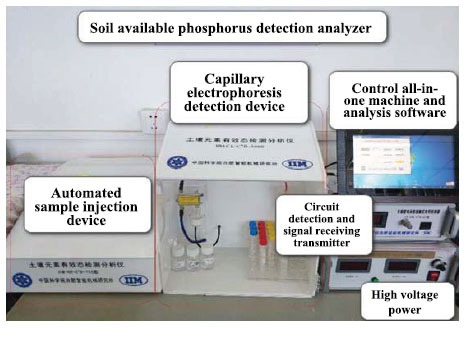
Soil available phosphorus detection analyzer.

The soil available phosphorus detection analyzer consists of the following parts:

1. Automated sample injection device offers automatic sampling injection and flushing pipelines for the device.
2. A capillary electrophoresis detection device is employed for sample handling and to maintain a constant temperature in the detection cell and capillary.
3. Control all-in-one machine and analysis software are used for process control and data analysis of detection analyzer.
4. Circuit detection and signal receiving transmitter are used for signal filtering, weak signal amplification, and sine wave transmission and reception.
5. High voltage power supply provides high voltage for sampling injection and separation (Fig. **4**).

## 4 RESULTS AND DISCUSSION

### 4.1 Standard Soil Sample Detection Test

The following standard samples were used: (1) Liaoning brown soil, (2) Henan yellow-tide soil, (3) Heilongjiang black soil, and (4) Anhui tide soil. The standard samples were analyzed using a soil available phosphorus detection analyzer and compared with the standard values. The following results were obtained.

**Fig. (5):**
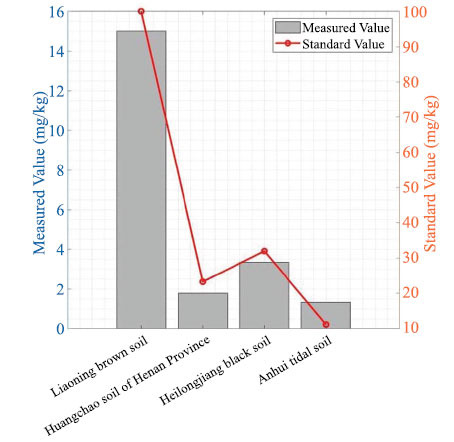
Comparison of actual available phosphorus test values and standard values in standard soil samples.

The detection values of the four soil standard samples showed consistent trends with the standard values, with only small errors, indicating that the soil available phosphorus detection analyzer can accurately detect phosphorus in standard soil samples (Fig. **5**).

### 4.2 Blind Test of Soil Samples

Using the SSQP-IIM-115 on-site soil rapid pretreatment with soil available phosphorus detection analyzer, flexible on-site rapid pretreatment and field detection of soil available phosphorus can be carried out. Random sampling points in Tai-he City, Anhui Province, obtained blind test values. Laboratory test standard values were obtained according to the National Ministry of Agriculture NY/T1121.7-2014 testing standards at the sampling points. The four sets of blind test values were compared with laboratory analysis values, with results shown inFig. (**6**)

Fig. (**6**) hows the blinded values agree well with the standardized values, with small errors and relative standard deviation RSD <3% for random samples.

The rapid soil pretreatment device faces limitations such as lower processing capacity, substantially below the 1-2 kg soil weight standards in labs. Compared to standard methods, it cannot independently correct system and human errors, so is primarily suitable for rough field estimates. However, the device offers unique contributions, such as utilizing ultrapure water for soil extractant in available phosphorus testing. Paired with a high-precision capacitively coupled contactless conductivity detector, it enables swift collection of soil phosphorus data in the field. This method is characterized by convenience, speed, eco-friendliness, and efficiency. The device's operation is user-friendly, employing data model algorithms for data analysis and processing, minimizing human error and ensuring accurate, swift acquisition of soil phosphorus data.

## 5 MATHEMATICAL MODELING AND THEORETICAL ANALYSIS

In the field of soil effective state capillary electrophoresis detection, we have conducted an in-depth study of the relationship between *V_1_* (volume of solution before spiking), *S_1_* (electrophoretic peak area before spiking) and *C_t_* (concentration of the sample solution to be tested) *via* mathematical modeling and machine learning techniques. The motivation for this research stems from the urgent need to optimize experimental procedures and gain a deeper understanding of ion content variation in soil samples. In terms of mathematical modeling, we adopt the polynomial fitting method [41, 42] to approximate the potential relationship between *V_1_*, *S_1_* and *C_t_* by flexibly adjusting mathematical functions. Polynomial fitting, as a traditional and reliable means of mathematical modeling, allows building concise and efficient mathematical models by adapting to data nonlinearity *via* simple polynomial functions. Simultaneously, we introduce machine learning methods including Support Vector Machine (SVM) [43], Gaussian Process Regression (GPR) [44] and Neural Networks [45]. These methods are more flexible and intelligent, accurately capturing complex *V_1_*, *S_1_* and *C_t_* relationships through learning large amounts of experimental data. SVM is renowned for excellent nonlinear modeling ability; GPR considers prediction uncertainty; neural networks achieve optimal nonlinear pattern fitting *via* multilevel learning. The significance of this research is optimizing experimental processes, reducing experiment number, and enhancing efficiency; while deepening understanding of ion content dynamics in soil samples; through mathematical modeling and machine learning. This provides smarter, advanced data analysis; means in soil science, and promotes deeper research and application. The integrated application of mathematical modeling and machine learning provides comprehensive, precise tools for experimental data interpretation in this evolving field.

### 5.1 Polynomial Fitting Model

Polynomial fitting is a common mathematical modeling method revealing variable relationships in experimental data *via* polynomial function fitting. In our study, we focused on the complex correlations between *V_1_* (solution volume before spiking), *S_1_* (electrophoretic peak area before spiking) and *C_t_* (concentration of sample solution to measure). The core idea is approximating experimental data by a polynomial function best reflecting actual observations. Our goal is to Our objective is to construct a polynomial model that reflects the effects of V1 and S1 on Ct by adjusting the polynomial coefficients. This intuitive mathematical model not only explains experimental data; and trends but also provides simple, intuitive analytical tools. The advantage of polynomial fitting lies in its simple, understandable form. By increasing the order of the polynomial, greater adaptability to data nonlinearities is possible, enabling the construction of relatively concise, efficient mathematical models. However, care must be taken not to introduce excessive complexity by over-fitting. In the current study, the application of polynomial fitting is crucial for explaining relationships between *V_1_*, *S_1_* and *C_t_*. Through mathematical modeling, interactions among factors can be better understood, supporting experimental design and data interpretation. In this research, we optimize experimental processes, enhanced efficiency, and contributed to the advancement of soil science. Polynomial fitting is a key tool, providing intuitive, effective modeling. It helps deeply explore *V_1_*, *S_1,_* and *C_t_* relationships, advancing soil science research. The fitting process is solved by least squares minimizing the sum of squared residuals of the fitted function from original data points, obtaining the best fitting function [46]:


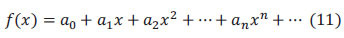


The fitting result is,


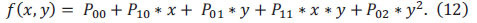


According to Equations 11 and 12, where is normalized by mean 1.371e+05 and std 1.221e+05, and is normalized by mean 36.3 and std 16.16; setting *S_1_* to *x*, *V_1_* to *y*, and *C_t_* to *f*(*x,y*).

Coefficients (with 95% confidence bounds):


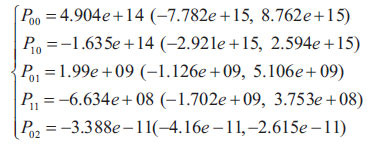


The model's goodness of fit for each parameter is: SSE: 77.91; R-square: 0.9154; Adjusted R-square: 0.9131; RMSE: 0.7231. Fig. (**7**) isualizes the polynomial fitting model.

### 5.2 SVM Regression Model

Support Vector Machine (SVM) is a powerful machine learning algorithm with applications in pattern recognition, classification, and regression analysis. SVM’s basic principle is to separate different sample classes by constructing a decision boundary. For regression, SVM aims to find a hyperplane minimizing the distance to sample points. The hyperplane’s location is determined by support vectors (points closest to the hyperplane). Therefore, the SVM effectively manages nonlinearities and exhibits good generalization. In our study, SVM models relationships between *V_1_*, *S_1_* and *C_t_*. By learning from large experimental datasets, SVM captures nonlinear *V_1_*, *S_1_* effects on *C_t_*, providing a flexible, intelligent modeling tool. Compared to traditional linear models, SVM demonstrates superior performance in addressing complex relationships. One key advantage of SVM is its potent modeling capability, which remains relatively insensitive to outliers. By incorporating a kernel function, SVM can map low-dimensional input spaces into higher dimensions. This enhances the capability for handling nonlinear relationships. The application of SVM not only improves modeling accuracy between *V_1_*, *S_1,_* and *C_t_*; but also helps uncover underlying patterns within experimental data. Specific modeling processes and results are detailed below [47-53].

Let the regression function in higher dimensional space be:


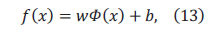


where *Φ*(*x*) is a nonlinear mapping function.


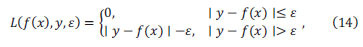


where *f*(*x*) is the SVM regression function prediction, *y* is the true value, and *ε* is a linear insensitive loss function.


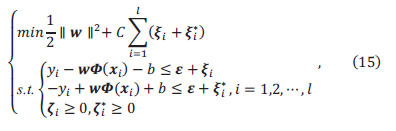


where *C* is the penalty factor and is the error requirement of the function. Introducing the Lagrangian function and transforming to dyadic form,


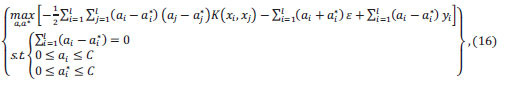


where *K*(*x_i_,x_j_*) is the kernel function.


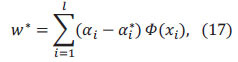



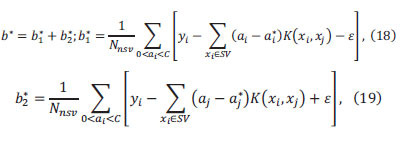


where *N_nsv_* is the number of support vectors.

The SVM regression function is,


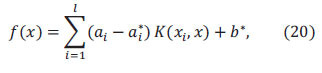


Figs. (**8** to **9**) show the visualized training results of the SVM regression model.

 Fig. (**8**) mainly shows the training state of the optimizable SVM model and the training effect, the left figure shows the error distribution of the model, and the right figure shows the minimum error iterative optimization of the model.

Fig. (**9**) mainly shows the degree of model fit and residuals, the left figure shows the degree of model fit, and the slope of the straight line has no practical significance and can only represent the size of the goodness-of-fit, while the right figure shows the distribution of the residuals of the model.

According to Eq. 13 to Eq. 20 as the specific steps of support vector machine regression, it can be seen that support vector machine regression can solve the regression problem well. And then the results from the statistical software show that the SVM regression model has a goodness of fit of 0.74.

### 5.3 Gaussian Process Regression Model

At the same time, we also introduce the Gaussian Process Regression (GPR) algorithm, a powerful nonparametric regression method, especially suitable for dealing with complex nonlinear relationships and unknown data distributions.

Compared to traditional methods, GPR not only provides prediction of the data; but also gives uncertainty of the prediction. It is based on modeling the a priori knowledge of the objective function and captures the relationship between input and output through a Gaussian distribution. In our study, we will utilize the GPR algorithm to establish a complex mapping relationship between electrophoretic peak area and solution volume.

The advantage of the GPR algorithm lies in its nonparametric nature, which can adapt to data distributions of various shapes; and is robust to noise. Through experimental data training, the GPR model will provide a flexible and adaptable mathematical model to better understand the dynamic changes between electrophoretic peak area and solution volume.

In practical applications, the introduction of the GPR algorithm enables more accurate prediction of electrophoretic peak areas for unknown samples and provides confidence estimates for these predictions. This provides a more accurate and reliable data analysis tool for detecting effective state capillary electrophoresis in soil; and more advanced and comprehensive methodological support for future soil science research development. The following is the specific modeling process [44, 54-60]:

The multivariate Gaussian distribution is shown in Eq.21:


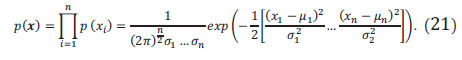


The vectorized representation of the multivariate Gaussian distribution is derived as in Eq.22:


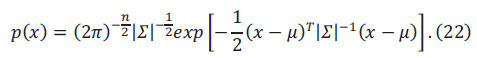


Assume the experimental dataset satisfies Eq.23:


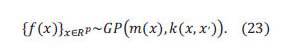


Here *m*(x) 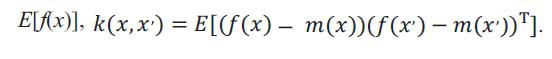


The prediction process for Gaussian regression is as follows:

Given the dataset as 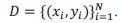



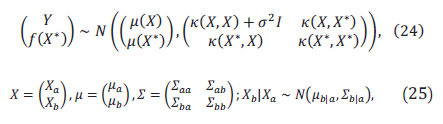


where,


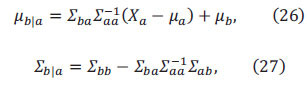


The final result obtained is:


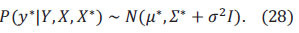


Eq. 21 to Eq. 28 are the specific steps of Gaussian regression, which can be predicted by solving the Gaussian regression function. Figs. (**10** and **11**) mainly show the training process and training effect of the Gaussian regression model. Fig. (**10**) mainly shows the training residual plot and minimum variance iteration plot of the model, from which you can gain an in-depth understanding of the specific training effect of the model. Fig. (**11**) shows the fitting effect of the model and the distribution of the residuals, and the fitting of the straight line has no practical significance, but just shows the effect of the model.

Finally, the results from the statistical software show that the Gaussian regression model has a goodness of fit of 0.82.

### 5.4 Neural Network Model

Neural networks are powerful machine learning algorithms for modeling complex nonlinear relationships and large-scale data. Compared to traditional methods, neural networks offer superior expressive and learning capabilities. By simulating interconnections between neurons in the human brain, they learn mapping relationships between inputs and outputs through multilayer networks. In this study, we utilize the neural network algorithm to establish a complex mapping between electrophoretic peak area and solution volume. The advantages of the neural network algorithm include its nonlinear fitting ability and adaptability to large-scale data. By training on experimental data, the neural network provides a highly flexible, adaptive mathematical model, better capturing underlying patterns between electrophoretic peak area and solution volume. In practical applications, neural network introduction allows for more accurate prediction of electrophoretic peak area in unknown samples, with the ability to handle large-scale datasets. This provides a more flexible, efficient data analysis tool for detecting effective state capillary electrophoresis in soil, and advanced, comprehensive methodological support for soil science research development. By integrating neural network fitting algorithms, we aim to achieve higher accuracy and intelligence in soil analysis [61-68].

Figs. (**12**-**14**) show the visualization of the training results of the neural network model. Fig. (**14**) reacts to the fitting curve of the final neural network model to the *C_t_* variable, which makes it easier for us to study the mathematical relationships implied between the variables.

The left panel of Fig. (**12**) shows the training state of the neural network model and the right panel demonstrates the error distribution of the neural network model.

The left panel of Fig. (**13**) shows the training performance of the neural network model, and the right panel demonstrates the training status of the neural network model for the test set, the training set, and the total goodness-of-fit.

Fig. (**14**) shows the fitted curves between the variables. The results from the statistical software show that the neural network model has a goodness of fit of 0.92.

By investigating relationships between *V_1_*, *S_1_* and *C_t_*, we used diverse modeling methods polynomial fitting, SVM, Gaussian regression, and neural networks to understand complex associations between these variables. The fitting results for each model are as follows: polynomial fitting is 0.9154, SVM is 0.74, Gaussian regression is 0.82, and neural network is 0.92.

In exploring the complex relationship between V_1_, S_1_, and C_t_, models such as polynomial fitting, support vector machine (SVM), Gaussian regression, and neural networks all exhibit unique advantages; but also present respective limitations. To accurately compare and evaluate these models, we must rigorously assess their characteristics and performances. First, polynomial fitting shows remarkable success in capturing nonlinear relationships with high goodness-of-fit (0.9154). Additionally, the polynomial model offers a concise mathematical form; and intuitively explains variable interactions, providing clear experimental data insights. However, improper order choice can lead to polynomial overfitting or underfitting, necessitating careful modeling consideration. Second, although the SVM model’s robustness is slightly lower (with a fit of 0.74), it is affected when dealing with nonlinearities. By separating data *via* optimal hyperplane identification in high-dimensional space, SVM proves resilient to noise and outliers. However, SVM performance hinges on kernel function and parameter selection, requiring optimization for specific problems. Gaussian regression falls between polynomial fitting and SVM in goodness-of-fit, reaching 0.82. Using kernel tricks, Gaussian regression maintains interpretability while enabling generalization. But compared to neural networks, Gaussian regression slightly underperforms complex pattern fitting, particularly with high-dimensional data and relationships. Finally, neural networks excel in capturing complex V_1_, S_1_, and C_t_ relationships, achieving up to 0.92 goodness-of-fit. Neural networks construct highly nonlinear models by neuron connection simulation, enabling powerful learning adaptation across complex data variations. They handle high-dimensionality and complexity robustly. However, neural networks are also prone to overfitting and face interpretability issues, necessitating cautious application. Comparing these four models comprehensively, each offers advantages fitting V_1_, S_1_, and C_t_ relationships. The polynomial fitting and neural network models demonstrated higher goodness-of-fit and greater accuracy in capturing complex relationships between variables. The SVM model exhibited superior robustness in handling nonlinear problems; Gaussian regression offered some generalization capability while maintaining a degree of interpretability. Nevertheless, each model has limitations and requires selection and optimization based on specific research aims and data characteristics. This study, synthesized these four models, providing methodological insights and empirical experience for soil science. By comparing fitting results and explanatory power across models, we deepened our comprehension of soil sample characteristics, furnishing lessons and insights for future related studies. Future investigations, could further explore advanced modeling methods like deep learning and integrated learning to more comprehensively reveal soil property complexity and diversity.

## CONCLUSION

This research successfully developed a rapid soil pretreatment device for field applications, along with an efficient protocol. Analysis of standard soil samples produced results closely aligned with established benchmarks, indicating high accuracy and validating the methodology's effectiveness. Integration of a sophisticated detection analyzer system enabled precise on-site measurement of soil available phosphorus. The system's precision was confirmed against standard soil samples, with results closely following anticipated trends, thus affirming its reliability. The SSQP-IIM-115 soil field pretreatment device, coupled with the soil-available phosphorus analyzer, effectively evaluated unknown soil samples. Compared to standard values per the Ministry of Agriculture's detection methods, the results exhibited a strong correlation, with a relative standard deviation (RSD) of less than 3%. Designed for field use, the equipment eliminates the need for additional power or water supplies. The portable system, suitable for vehicle mounting, conducted pretreatment and testing directly at sampling sites, minimizing environmental contamination. With minimal maintenance requirements and rapid data upload capability, the system was well-suited for large-scale rapid soil surveys.

The development and utilization of the rapid soil pretreatment device underscored the feasibility of conducting on-site soil analysis, offering a dependable approach for soil quality assessment. The validation of the detection analyzer system through testing with standard soil samples confirmed its accuracy and reliability in soil phosphorus detection, providing a robust solution for environmental monitoring. The combined use of the SSQP-IIM-115 device and the phosphorus analyzer demonstrated efficacy in determining soil available phosphorus levels on-site, boasting high accuracy and reliability as evidenced by the low relative standard deviation. With its portability and self-sufficiency, the system was ideally suited for comprehensive soil surveys across diverse environments, including those with limited resources. The utilization of advanced mathematical modeling techniques, such as polynomial fitting, Support Vector Machines (SVM), Gaussian regression, and neural networks, greatly deepened comprehension of soil component interactions. Notably, the neural network model exhibited outstanding performance in fitting complex data, underscoring its promise as a predictive tool in soil analysis and environmental science.

As a perspective, we note that the advancement of portable detection systems is also a crucial focus, especially in agricultural and food safety contexts. A portable system for on-site soil pH and potassium detection, utilizing 3D printed sensors and a PSoC4 microcontroller, eliminated the need for lab equipment and pre-treatment. Paper-based lab-on-a-chip (pLOC) devices offered rapid detection of contaminants using various fabrication methods and detection techniques. Integration of ICT tools enhanced real-time monitoring and traceability in the food supply chain. Portable pLOC devices provided rapid on-site detection of contaminants and could be integrated with smartphones. It is anticipated that the techniques developed in this work will also benefit portable detection systems.

## Figures and Tables

**Fig. (1):**
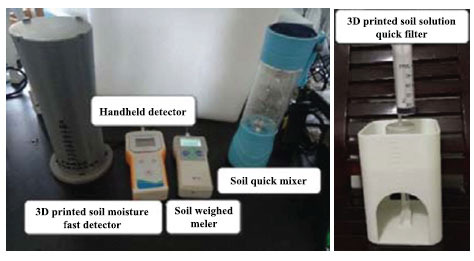
SSQP-IIM-115 on-site soil rapid pretreatment device.

**Fig. (2):**
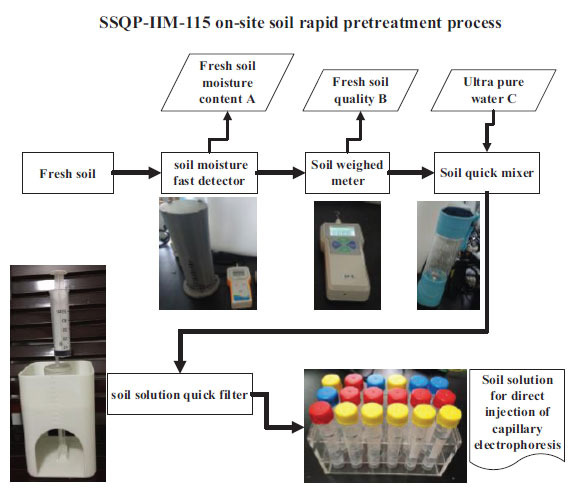
SSQP-IIM-115 on-site soil rapid pretreatment process.

**Fig. (3):**
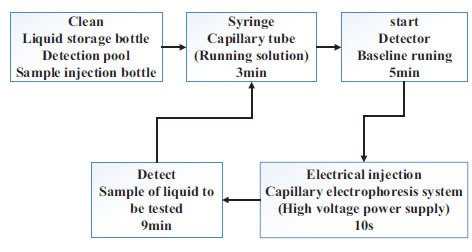
Capillary electrophoresis capacitively coupled contactless conductivity detector operating process.

**Fig. (6):**
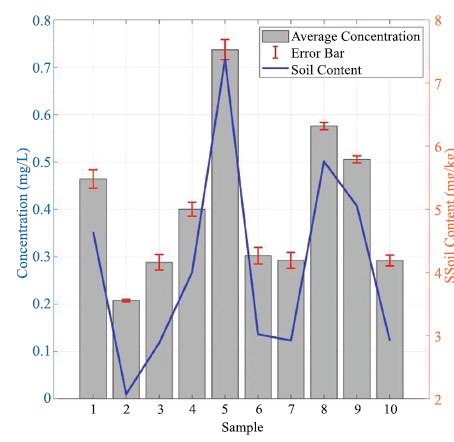
Comparison of blind measurements of soil available phosphorus with standard values and relative standard deviations in Tai-he County, Anhui Province, China.

**Fig. (7):**
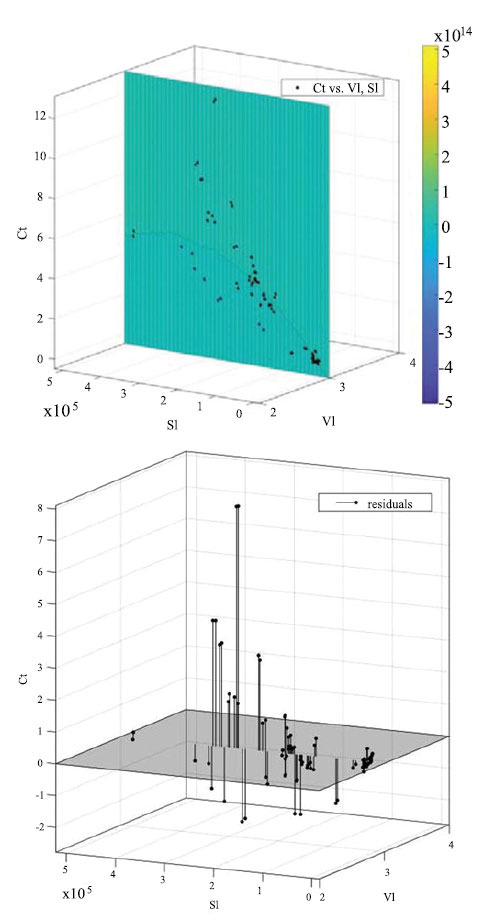
Polynomial fitting visualization with model residual distribution.

**Fig. (8):**
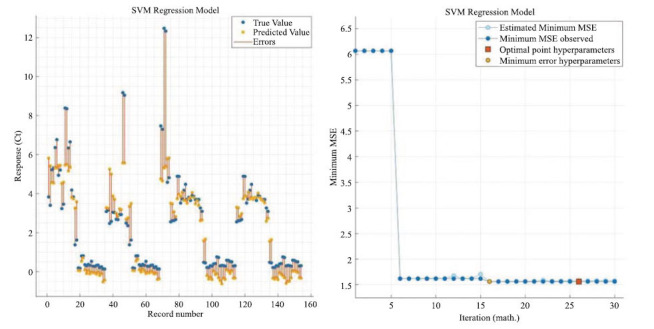
SVM regression model response with minimum error iteration.

**Fig. (9):**
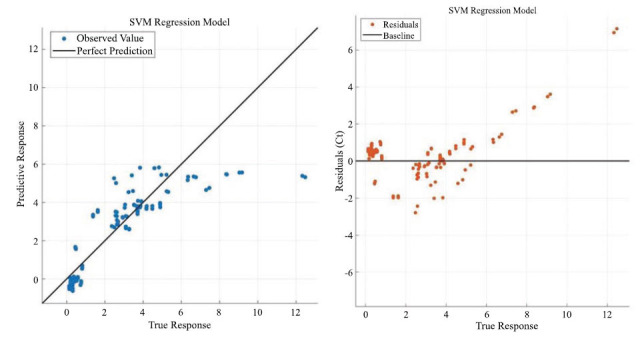
SVM regression model fitting regression with residual distribution.

**Fig. (10):**
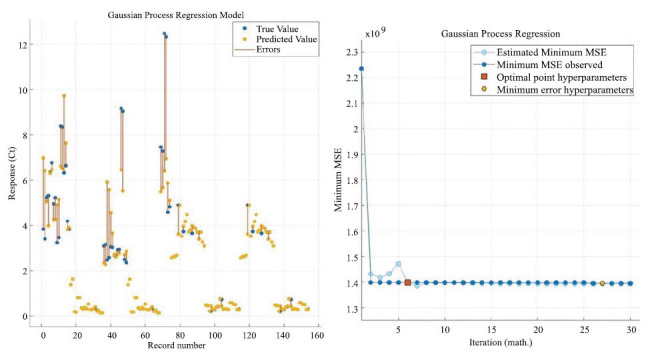
Gaussian process regression model response with minimum error iteration.

**Fig. (11):**
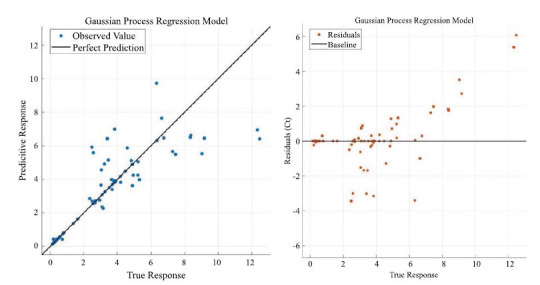
Gaussian process regression model fitting regression with residual distribution.

**Fig. (12):**
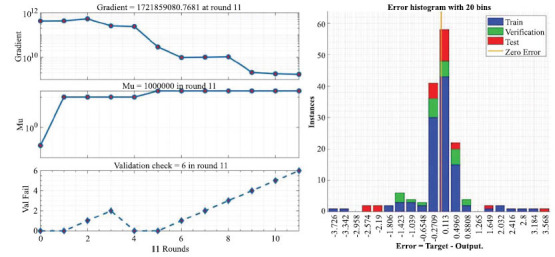
Neural network training state and residual distribution.

**Fig. (13):**
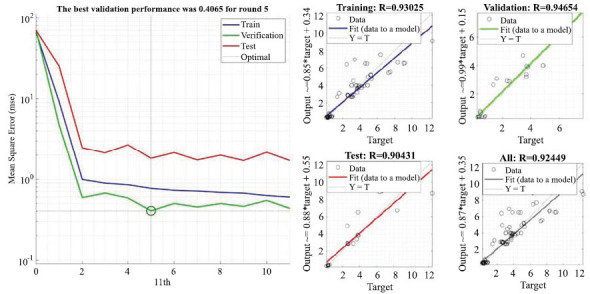
Neural network model performance with fitted regression.

**Fig. (14):**
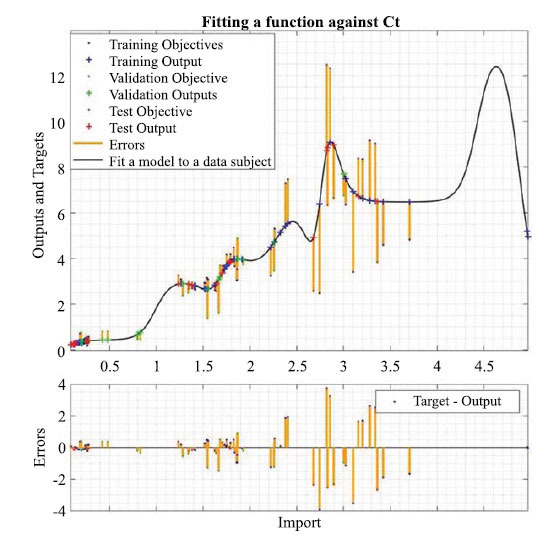
Visualization of the fit of the neural network model to the *C_t_* variable.

## Data Availability

The data and supporting information are provided within the article.

## LIST OF ABBREVIATIONS

3D printed: Three-Dimensional printed
AC signal: Alternating Current signal
C4D: Capacitively Coupled Contactless Conductivity Detection
GPR: Gaussian Process Regression
HP-β-CD: Hydroxypropyl-β-cyclodextrin
RMSE: Root Mean Square Error
RSD: Relative Standard Deviation
SSE: Sum of Squares for Error
SSQP-IIM-115: System of Soil Quick Pretreatment-Institute of Intelligent Machine-115
SVM: Support Vector Machine
